# Identification of the size and location of dyssynchronous regions in patients undergoing CRT

**DOI:** 10.1186/1532-429X-15-S1-P71

**Published:** 2013-01-30

**Authors:** Jonathan Suever, Richard P Magrath, Michael Lloyd, John N Oshinski

**Affiliations:** 1 Wallace H. Coulter Department of Biomedical Engineering, Georgia Institute of Technology / Emory University, Atlanta, GA, USA; 2 Division of Cardiac Electrophysiology, Department of Medicine, Emory University School of Medicine, Atlanta, GA, USA; 3 Department of Radiology and Imaging Sciences, Emory University School of Medicine, Atlanta, GA, USA

## Background

It has been shown that both the degree of LV mechanical dyssynchrony and the location of the latest contracting segment of the LV affect an individual's ability to respond positively to CRT [Bax et al JACC 2005]. From high temporal resolution short-axis SSFP images, maps of regional mechanical contraction times throughout the LV can be generated. These maps show regional dyssynchrony and can be used to assist in CRT lead placement planning.

The objective of this study was to: 1) characterize the mechanical contraction times typically observed in a healthy population and determine threshold values for normal contraction times and 2) apply these thresholds in a set of patients enrolled for CRT to determine the size and location of areas of late mechanical contraction (dyssynchronous regions).

## Methods

Cardiac MR exams were performed on 10 healthy volunteers with no evidence of mechanical dyssynchrony (QRS < 120ms) and in 27 patients scheduled for CRT that met current inclusion criteria.

**Figure 1:**
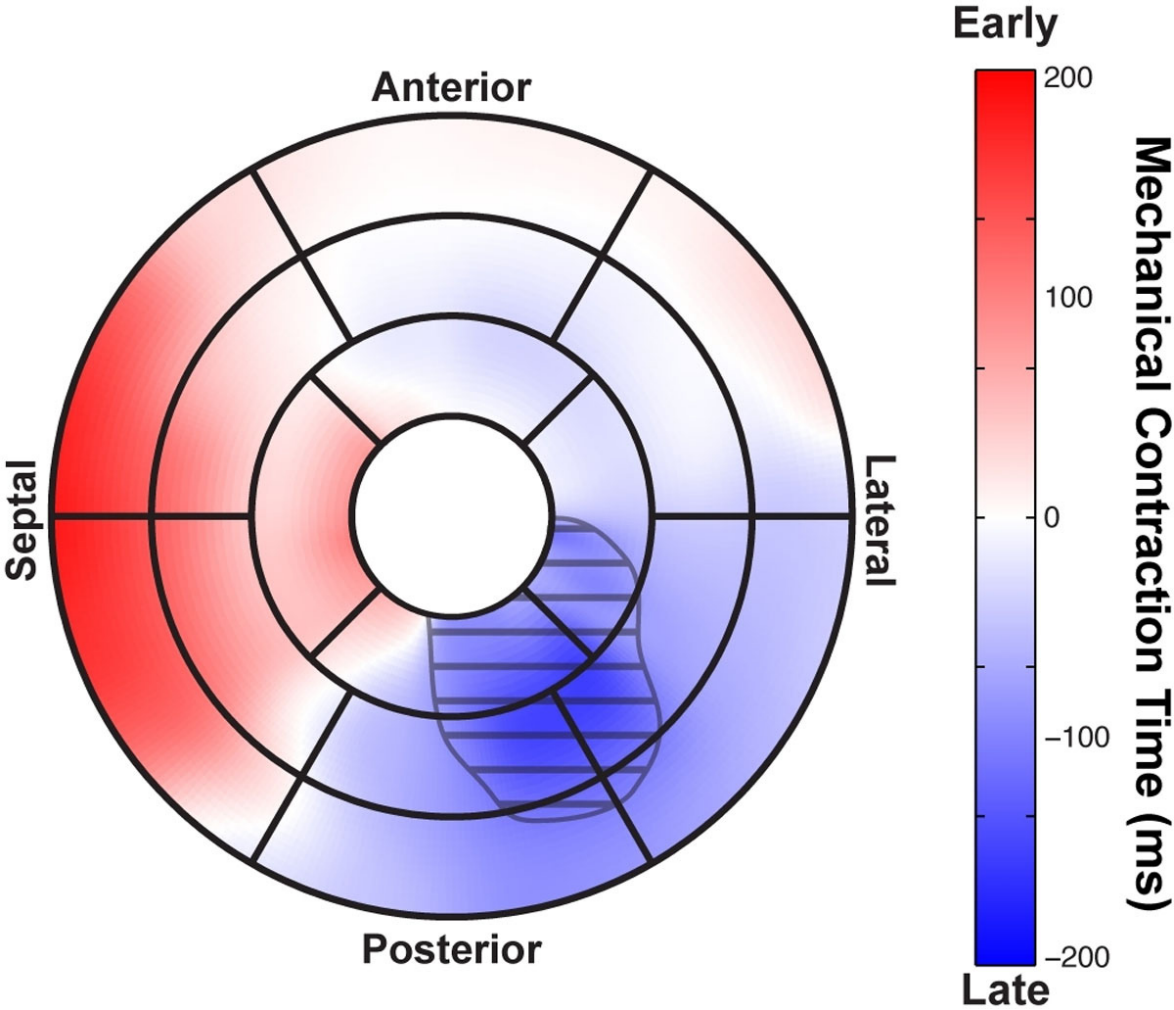
AHA 17-segment model showing regional contraction times. The shaded area was identified as a dyssynchronous region.

MR cine short-axis SSFP images were acquired with 60 frames per cardiac cycle. Radial shortening at 100 locations was determined for each slice. Mechanical contraction times were computed by finding the temporal delay between the radial motion curve at each location relative to a patient-specific reference. A map of regional mechanical contraction times for each patient was generated and projected onto an AHA 17-segment bullseye (Figure 1).

Limits of "normal" contraction were defined using a 95% confidence interval based on delay times for all healthy volunteers. By comparing delays seen in each patient to the "normal" contraction, we identified dyssynchronous regions of the LV, the size of the dyssynchronous regions (shaded region in Figure 1).

## Results

**Figure 2:**
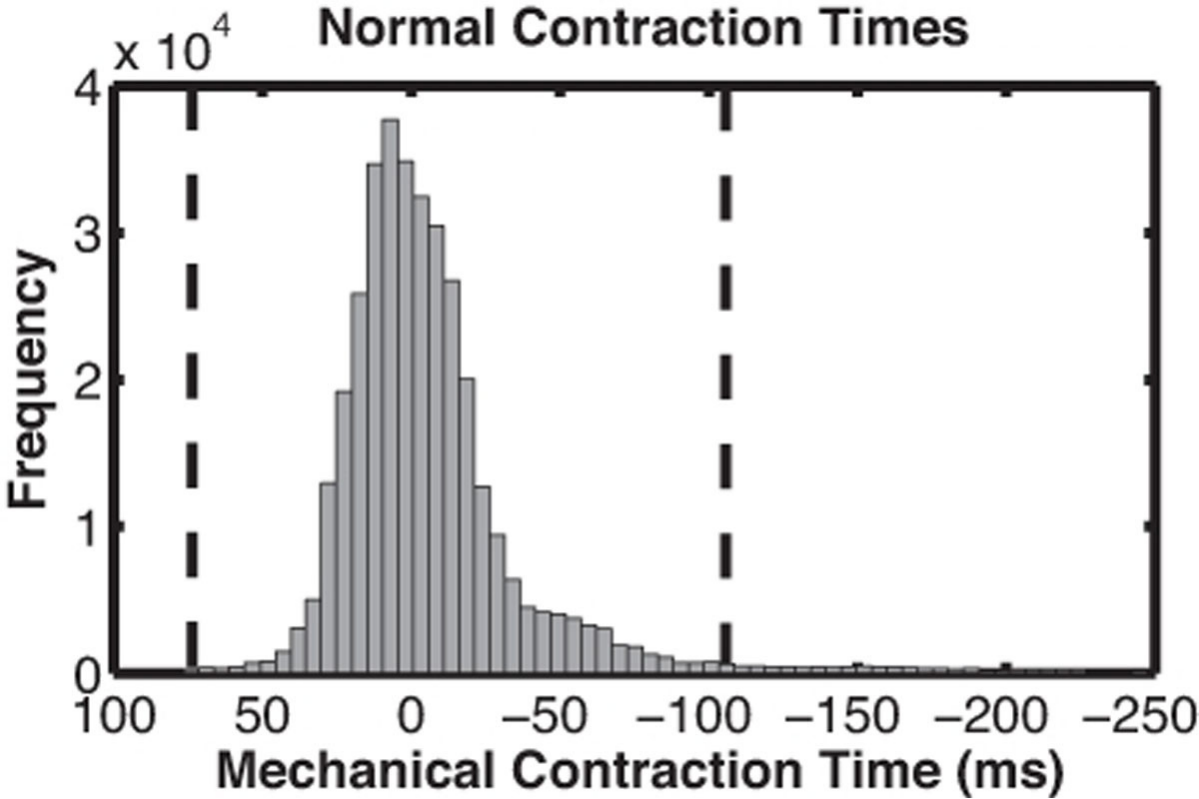
Distribution of contraction times from all healthy volunteers.

Healthy volunteers had a mean mechanical delay time of -10.8 ± 47.4ms. Using the 95% confidence interval, "normal" mechanical delay times were determined to lie in the range: -103.7 - 82.1 ms (Figure 2).

CRT patients had 2.4 ± 3.6 dyssynchronous segments making up 36.4 ± 11.9 % of the LV.

The location of the most dyssynchronous region was found to be septal in 7 patients, posterolateral in 8 patients, and anterolateral in 12 patients. The variety seen in the location of the latest contracting segment agrees with the results of the MADIT-CRT study that found significant heterogeneity in the location of dyssynchronous segments in CRT patients.

## Conclusions

Regional mechanical dyssynchrony maps were computed from high temporal resolution cine CMR images for both healthy volunteers and patients enrolled for CRT. Mechanical delay times in patients were compared to normal values to find regions of dyssynchrony. The location of these dyssynchronous regions varied significantly.

## Funding

Funding for this research was provided by AHA Grant-in-Aid, the National Science Foundation Graduate Research Fellowship Program, and by the National Center for Advancing Translational Sciences of the National Institutes of Health Award Number UL1TR000454.

